# Visual Puzzles, Figure Weights, and Cancellation: Some Preliminary Hypotheses on the Functional and Neural Substrates of These Three New WAIS-IV Subtests

**DOI:** 10.5402/2011/123173

**Published:** 2011-08-23

**Authors:** Simon M. McCrea, Thomas P. Robinson

**Affiliations:** ^1^Department of Neuropsychology, Wascana Rehabilitation Centre, 2180—23rd Avenue, Regina, Saskatchewan, Canada S4S 0A5; ^2^Functional Rehabilitation Program, Wascana Rehabilitation Centre, 2180—23rd Avenue, Regina, Saskatchewan, Canada S4S 0A5

## Abstract

In this study, five consecutive patients with focal strokes and/or cortical excisions were examined with the Wechsler Adult Intelligence Scale and Wechsler Memory Scale—Fourth Editions along with a comprehensive battery of other neuropsychological tasks. All five of the lesions were large and typically involved frontal, temporal, and/or parietal lobes and were lateralized to one hemisphere. The clinical case method was used to determine the cognitive neuropsychological correlates of mental rotation (Visual Puzzles), Piagetian balance beam (Figure Weights), and visual search (Cancellation) tasks. The pattern of results on Visual Puzzles and Figure Weights suggested that both subtests involve predominately right frontoparietal networks involved in visual working memory. It appeared that Visual Puzzles could also critically rely on the integrity of the left temporoparietal junction. The left temporoparietal junction could be involved in temporal ordering and integration of local elements into a nonverbal gestalt. In contrast, the Figure Weights task appears to critically involve the right temporoparietal junction involved in numerical magnitude estimation. Cancellation was sensitive to left frontotemporal lesions and not right posterior parietal lesions typical of other visual search tasks. In addition, the Cancellation subtest was sensitive to verbal search strategies and perhaps object-based attention demands, thereby constituting a unique task in comparison with previous visual search tasks.

## 1. Introduction

The Wechsler Adult Intelligence Scale—Fourth Edition released in 2008 is the most current edition of the Wechsler Intelligence Scales dating from the initial Wechsler-Bellevue first published in 1939 [1]. Loring and Bauer note that the conormed Wechsler Adult Intelligence Scales and the Wechsler Memory Scales are the two most common psychological tests used in clinical care and research in neurology [2]. There have been significant structural and content changes in the Fourth Edition. These changes include the addition of three new subtests of Visual Puzzles, Figure Weights, and Cancellation as well as the deletion of Object Assembly and Picture Arrangement from the WAIS-III. Added benefits for this new instrument include lower floor items and much higher difficult ceiling items, thereby providing greater measurement stability at lower and higher ability levels. Loring and Bauer cautioned that there are presently insufficient data on neurological populations to ensure appropriate application of the WAIS-IV for neuropsychological evaluations. While we agree with Loring and Bauer on this point, there are still 12 subtests that aside from some new content additions and updates are largely unchanged. This study is an effort to integrate and synthesize the extant data on the cognitive psychological, neuropsychological, and functional neuroimaging literatures on the correlates of the three new tasks of Visual Puzzles, Figure Weights, and Cancellation. 

Benson and colleagues noted in their 2010 article that the WAIS-IV standardization data showed that the Cattell-Horn-Carroll- (CHC-) theory based structure provided an excellent description of this sample's performance. These authors further postulated that the CHC theory perhaps provided a better interpretation than the published scoring structure does [1]. The Cattell-Horn-Carroll model of intelligence resolves discrepancies between Carroll's three-stratum theory of general intelligence [3] and Cattell's original fluid and crystallized model of human cognitive capabilities. McGrew's CHC theory accommodates a large amount of psychometric data accruing from these two influential and contemporary models of the structure of human abilities [4]. CHC theory is a hierarchical model of human abilities with three strata. The general factor which has been shown to account for the broad positive correlations among all mental tasks is at the apex and constitutes stratum III. Stratum II consists of 10 broad abilities. In the WAIS-IV, these abilities include measures of crystallized intelligence (G*c*), fluid reasoning (G*f*), general spatial visualization (G*v*), short-term memory (G*sm*), and processing speed (G*s*). The WAIS-IV also provides a measure of quantitative reasoning (QR) at Stratum II unlike other intelligence tests. Stratum I consists of over 100 narrow CHC abilities [5] that are beyond the scope of this paper.

In the Visual Puzzles subtest, examinees are required to view a completed puzzle and select three elements from 6 stimulus shapes that can be combined in their mind's eye via edge completion to form the gestalt puzzle. In the Figure Weights subtest quantitative reasoning skills are required to select the appropriate missing weights needed to balance a scale. The Figure Weights subtest is similar in design to Piagetian balance beam tasks [6]. Finally, the new Cancellation subtest requires examinees to distinguish the color and shape of stimuli and identify target shapes via manual crossing through of items. Benson and colleagues showed that the Figure Weights, Visual Puzzles, and Cancellation's loadings on the general factor were 0.78, 0.68, and 0.37, respectively [1]. Figure Weight's g loading was the highest of all of the 15 WAIS-IV subtests and the next highest loading subtests included Arithmetic (0.75), Vocabulary (0.74), and Similarities and Block Design (both at 0.70). The lowest loading subtest in the WAIS-IV is Cancellation at 0.37, and it is construed as primarily a measure of processing speed. The Cancellation subtest should theoretically also constitute a good means of assessing visual field neglect. Visual field neglect can adversely affect any number of higher-order cognitive functions. Also of interest was that Block Design has been supplanted by Figure Weights in the WAIS-IV as the highest loading nonverbal subtest.

### 1.1. Visual Puzzles

The Wechsler Adult Intelligence Scale—Fourth Edition's Visual Puzzles is most similar in structure and tapped cognitive processes to the Minnesota Paper Form Board [7]. The Minnesota Paper Form Board (MPFB) requires mental transformation, manipulation, and analysis of dimensional objects. The test uses geometric shapes to assess the examinee's mechanical-spatial abilities. The original MPFB test consisted of 64 two-dimensional shapes that were drawn in separate pieces, requiring the examinee to visualize the pieces as a complete geometric shape in order to derive the correct answer. In the Visual Puzzles subtest, the examinee views a completed puzzle and selects three options that when combined in the mind's eye reconstruct the puzzle. Visual Puzzles is a new subtest designed to measure nonverbal reasoning and the ability to analyze and synthesize abstract visual stimuli [8].

Neuroimaging data have shown activation of the cerebellum during the mental rotation of objects [9], and Molinari et al. found that left cerebellar patients were especially prone to unsuccessfully complete items from the Minnesota Paper Form Board [10]. The left cerebellar localization given decussation of frontocerebellar tracts suggests a predominance of right hemisphere cortical processing on the MPFB [11]. Molinari et al. note that the Block Design subtest of the WAIS is similar to the MPFB, since a geometric visuospatial design is used as a template to which segments must be matched. A nonverbal figure is used as the stimulus in both tasks and both tests require rotation of the stimulus components [10]. However, the Block Design subtest can be solved by direct manipulation of the component blocks using chance associations and contour completion, while the MPFB can only be solved by mentally rotating the parts. Furthermore in the Block Design subtest, subjects are required to match each block directly with the solution template, whereas in the MPFB, direct comparison is impossible. These authors note that in the MPFB test, subjects must assemble the stimuli mentally and then compare the mentally assembled figure with five possible solutions. Molinari et al.'s data suggests that cerebellar damage may affect the ability to undertake such visuospatial manipulations mentally.

Linn and Petersen observed that the MPFB can be solved using both visual and verbal strategies even when rotating of components is required [12]. Voyer et al.'s meta-analysis of factor analytic studies showed the MPFB primarily measures general spatial visualization [13]. General spatial visualization is exemplified by paper-folding tasks which are close to the apex of stratum III along with archetypal tests of fluid intelligence like the Raven Progressive Matrices [3]. Paper folding is a three dimensional analog of the Minnesota Paper Form Board test which can be administered in verbal and nonverbal formats. Large between-subject differences in general spatial visualization have been repeatedly demonstrated [3]. The WAIS-IV is among only a handful of intelligence tests that possess the capability to assess this ability at the upper and lower extremes of the normal distribution. 

Bilateral posterior parietal cortex as well as regions extending down into the posterior higher-order visual cortices are consistently activated by mental rotation across a range of tasks, types of neuroimaging techniques, or statistical analysis procedures [14]. Functional neuroimaging findings are consistent with older neuropsychological lesion studies [15] and transcranial magnetic stimulation studies of mental rotation [16]. It appears conclusive from these studies that mental rotation involves analog spatial representations, since the time for mental rotation is linearly related with response time [17]. Zack's review also found that motor areas of the posterior frontal cortex were clearly activated bilaterally during mental rotation. These modulations in the precentral cortex appear to involve motor simulations of such representational rotation.

### 1.2. Figure Weights

In the Figure Weights task, an examinee views scales with missing weights and selects the response option that is best suited to keep the scales balanced. It is reported in the WAIS-IV Technical Manual that the Figure Weights task measures quantitative and analogical reasoning [8]. These types of Piagetian tasks routinely assess quantitative reasoning that can be expressed mathematically through deductive and inductive logic [3]. Arguably, Figure Weights is most similar to Piagetian balance beam tasks [6] although in the original Piagetian tasks on which Figure Weights was modeled, the examinee was often required to simultaneously integrate weight data with proportional distance from the fulcrum. Figure Weights in contrast only uses different colors and shape tokens as weights without the necessity of incorporating proportional distance from the fulcrum. This would be the prime difference between Figure Weights and the original Piagetian tasks. In this sense, the WAIS-IV's Figure Weights subtest is a significant and unique innovation and may have few if any parallels in the prior psychometric literature.

The balance beam test is a well-known Piagetian task [18], and successful performance on it has been classically considered to be an indicator of the development of formal stage of operations. In his review, Carroll notes that Piagetian reasoning tasks correlate most highly with the general intelligence factor and then with fluid intelligence factor and subsequently with crystallized intelligence [3]. de Ribaupierre and Lecerf found that working memory explained more than 80% of the age-related variance in performance on these types of Piagetian tasks in adults [6]. These developmental psychologists have shown that Piagetian tasks are relatively pure measures of fluid intelligence and are relatively less influenced by knowledge base and strategies. Recent psychometric studies showed that Figure Weights is the highest loading fluid intelligence subtest in the WAIS-IV battery and that it can be best described as a combination of quantitative reasoning and fluid intelligence [1]. A quantitative reasoning composite score on the WAIS-IV was further characterized in a 1 : 1 equation of Figure Weights and the Arithmetic subtests by these authors [1].

Although there are at present no direct prior published neuropsychological tests that closely correspond with the cognitive processes used in performance on the Figure Weights subtest, a decomposition of the task parameters suggests a high loading of fluid intelligence. The high loading on fluid intelligence suggests greater reliance on frontal-mediated executive attention processes such as online monitoring and decision making. Fluid intelligence has been shown to be dependent on a network of regions including the lateral prefrontal cortex, posterior parietal cortex, dorsal anterior cingulate and the lateral posterior cerebellum involved in executive attentional control [19]. Recall that fluid intelligence is just one of two factors that Figure Weights loads on including quantitative reasoning. Although Arithmetic is a verbal quantitative reasoning task other more recent neuropsychological studies have shown that mathematical reasoning can be completely independent of language at least in adults. Varley et al. (2005) showed that a diverse spectrum of mathematical reasoning processes remained intact in patients with large language-dominant hemisphere lesions [20].

It is hypothesized that neural networks that instantiate syntax for language acquisition in the linguistically dominant left hemisphere can also potentiate mathematical reasoning development in the intact right hemisphere [20]. The publication of cases of three adult subjects with large left hemisphere lesions and intact mathematical reasoning cognitive profiles were incompatible with the claim that mathematical expressions are first translated into a linguistic format [20]. It has been previously hypothesized that mathematical principles, rules, and reasoning must first gain access to syntactic mechanisms specialized for language in the left hemisphere in a bottleneck manner [20]. The importance of the language-dominant hemisphere in the performance of advanced mathematical reasoning is a truism that is often taken as a given in studies of acalculia, anarithmetria and mathematical disability. 

And as Haavist and Lehto note, verbal and spatial working memory are highly correlated with crystallized and fluid intelligence, respectively. Haavist and Lehto's assertion implies a verbal/crystallized/left hemisphere and spatial/fluid/right hemisphere parcellation into opposing hemispheres [21]. Finally, in a review, Gathercole noted that components of Baddeley's visuospatial sketchpad consisting of spatial working memory/fluid intelligence are routinely adversely affected by lesions of the inferior prefrontal cortex and posterior parietal cortex in the right hemisphere [22]. In such functional neuroimaging studies, the lateral occipital complex involved in object recognition and mental representation of visuospatial percepts are sometimes also activated within the right hemisphere during spatial working memory tasks [22]. On the basis of these findings, it would be hypothesized that Figure Weights would be reliant on the dorsal frontoparietal circuits within the right hemisphere.

### 1.3. Cancellation

Cancellation is a supplemental subtest that requires that subjects scan a structured arrangement of colored shapes and mark the targets and avoid the distractors. This subtest is similar to cancellation tasks designed to measure processing speed, visual selective attention, vigilance, perceptual speed, and visuomotor ability. Such cancellation of lines or 2-dimensional letters across the left and right visual fields have been used for many years in clinical neuropsychology [8]. Cancellation tasks are often used to assess visual neglect, response inhibition, and motor perseveration. The wide angle horizontal field of view of the WAIS-IV's Cancellation subtest would render it suitable for examining unilateral spatial neglect in the left and right visual fields if necessary. There are numerous types of cancellation tasks. Halligan and Marshall showed that the Test of Visual Neglect was able to parse two dimensional neglect [23]. Not only was left neglect evident with right cerebrovascular accident (CVA) but also there was a graded selective inattention within each of the four sections of the visual field. Similarly, the Bells Test identified a significant number of visual neglect patients suffering from right CVA [24]. Both of these tests are similar to the WAIS-IV's Cancellation subtest.

Neglect characteristically involves a failure to acknowledge or explore stimuli towards the contralesional side of space. Visual neglect is usually more frequent and severe after right hemisphere lesions [25, page 481], and it often co-occurs with the complicating factor of anosognosia. In an early study Hecaen described unilateral spatial neglect after 31% of right hemisphere lesions (total sample = 176 right hemisphere cases) and in only 1 out of 276 cases with left hemisphere damage [26]. The isolated reversed case would presumably result from some type of crossed or mixed dominance of language and spatial functions [27]. Left visual neglect is routinely associated with stroke lesions of the right inferior parietal lobe and/or the right temporoparietal junction [28, 29]. More recently, Urbanski and colleagues showed that visuospatial neglect correlated with specific damage to the frontoparietal and fronto-occipital white matter fiber tracts in the right hemisphere. These fasciculi connect the dorsal spatial attentional neural substrates of the inferior parietal lobule with the premotor and prefrontal cortex. These diffusion tensor imaging studies show that chronic visual neglect can result from, and correlate with, damage to frontoparietal connections in the right hemisphere presumably involved in spatial working memory [30].

## 2. Materials and Methods

In this study, five subjects with well-characterized stroke and/or surgical resection lesions were referred by physiatrists, neurologists or neurosurgeons, for comprehensive neuropsychological evaluations. The patients were consecutively recruited into the study over an eight-month period (April of 2010 to January of 2011) at the Wascana Rehabilitation Centre in Regina, Canada. This neuropsychological study was provided with consent to proceed by Research and Performance Support of the Regina Qu'Appelle Health Region in 2010. All patients were informed about the study and gave their verbal permission and signed an informed consent form prior to participation. The sample consisted of two women and three men with four right-handers and one left hander. All subjects had sustained focal singular ischemic and/or hemorrhagic stroke and/or neurosurgical resections that were unambiguously able to be delineated on either CT or MRI scans. Subjects were assessed with the entire WAIS-IV [31], WMS-IV [32], and Advanced Clinical Solutions Social Perception Battery [33]—see Table 1. These core batteries were supplemented by tests of attention, executive, motor, and sensory functions.

The mean age of the sample was forty-three (SD = 16), and the group as a whole was more highly educated than the general population. All subjects had completed some college courses or advanced trades postsecondary training with a mean level of education of 15 years (SD = 2). Similarly the premorbid FSIQ as estimated using the Advanced Clinical Solutions Test of Premorbid Functioning was above average for the group (*X* = 112, SD = 8). The mean duration that subjects were assessed was 14 months after stroke (SD = 11). Hence, the subjects were assessed approximately one year poststroke suggesting that all subjects had experienced near asymptotic levels of functional recovery [34]. Finally, as an index of the severity of these patient's strokes, the average obtained FSIQ was found to be noticeably attenuated at 84 (SD = 13). 

Thus, these subjects had as a result of their rather large strokes and/or cortical excisions suffered a minimum one standard deviation unit loss in general intellectual functioning. This large effect of the brain injuries fits well with a neuropsychological methodology of using “critical threshold lesions” that would be expected to result in disruption of essential functional neuroanatomical pathways and/or attenuation of specific cognitive functions. Also, three of the subjects had right hemisphere lesions (right frontotemporal lobe, right posterior thalamus, and right temporoparietal lobe) and two of the subjects had left hemisphere lesions (left temporoparietal lobe and left prefrontal lobe). The mean obtained Verbal Comprehension Index (VCI) score was 85 (SD = 25) and the mean obtained Perceptual Reasoning Index (PRI) was 82 (SD = 17). The near parity between the VCI and PRI would be consistent with the assertion that the cumulative performance attenuating effects of these brain lesions were similar for the left and right hemisphere injury participants.

Five participants constitutes a small sample and a group mean approach with sufficient statistical power (e.g., ≥8 subjects per lesion group) might constitute a better characterization of the nature of neural damage effects on performance on the WAIS-IV subtests. However, the cognitive neuropsychological approach is not without its unique strengths [35]. Double dissociations between subtests as well as the overall neuropsychological profile of each individual case can be used to develop a first approximation of the neural substrates of performance on these new WAIS-IV subtests. This strength lies in the methodology of using the “configuration of signs” rather than isolated test signs in pointing towards specific syndromes [35]. When stroke lesions are large enough and of sufficient magnitude to cause focal neuropsychological syndromes—an *impaired/normal* qualitative approach to lesion characterization should enable delineation of neural systems involved in performance. In addition, the use of an overarching neuropsychological theoretical orientation or rationale can obviate the need for large samples when determining the neural correlates of performance on any battery [35].

This paper will be guided by the influential two cortical streams theory of Milner and Goodale which posits higher-order cognitive functions associated with specific neural tracts and pathways. The theory is increasingly gaining credence as a first-generation model of consciousness framed entirely in plausible neuropsychological systems [47]. The dorsal stream or “where pathway” is involved in mapping spatial locations of stimuli. It begins with primary visual cortex and traverses upwards towards the posterior parietal cortex and then forward towards the dorsal prefrontal cortex. The where pathway maps spatial locations and the control of the eyes and hand when visual information is required for guiding saccades or when reaching for objects. The ventral stream or “what pathway” is involved in mapping the identity of stimuli. It similarly begins with primary visual cortex but traverses downwards through the inferior temporal cortex and forward towards the temporal pole via the longitudinal fasciculus. High-level object representations constructed in the anterior temporal lobe thus presumably travel forward towards the orbitofrontal cortex via the uncinate fasciculus. The what pathway maps form recognition and object representations. 

Milner and Goodale's model stands alone among comprehensive neuropsychological theories of higher cortical functions and contends that most human cognitive functions can be interpreted in terms of these parameters [47]. Unlike prior complete theories of higher cortical functions like Alexander Luria's of the 1960s and 1970s [48] Milner and Goodale's theory incorporates a wealth of neuropsychological, electrophysiological, functional neuroimaging, neuroanatomical, and behavioural data. This contemporary and compelling large body of empirical research using both humans and primates views the human brain as composed of multiple redundant and re-entrant neural networks that are highly parallel in nature. Moreover, according to the theory, there are at least four main pathways or sequences of successive fascicular connections that in essence constitute the “neural tracts instantiating cortical processing” (e.g., left hemisphere's verbal dorsal/ventral streams and right hemisphere's spatial dorsal/ventral streams). 

These pathways are present in all human brains, yet some evidence suggests that one or more such pathways may predominate within individuals based on variables such as gender, language localization, and/or spatial reasoning localization [49]. Newer techniques in cognitive neuropsychology as well as structural neuroimaging methods developed in the last decade (such as diffusion tensor MRI) may be capable of characterizing the neural basis of such individual differences. More recent models of higher cortical function stress the importance of secondary bridging of dorsal and ventral cortical streams emanating from the superior parietal and anterior temporal cortex to the dorsolateral prefrontal and inferior prefrontal cortex, respectively [49]. These new white matter fiber tract neuroimaging studies show coextensivity and compatibility of Milner and Goodale's model of posterior cortical function with current models of prefrontal cortex function [50]. Other statistical techniques such as Granger causality mapping and magnetoencephalography with a millisecond-level grain of resolution may be capable of developing specific box-models of the structural and temporal sequence of such large-scale brain systems.

## 3. Results

### 3.1. Participant 1: Left Temporoparietal Lesion

**Figure 1:**
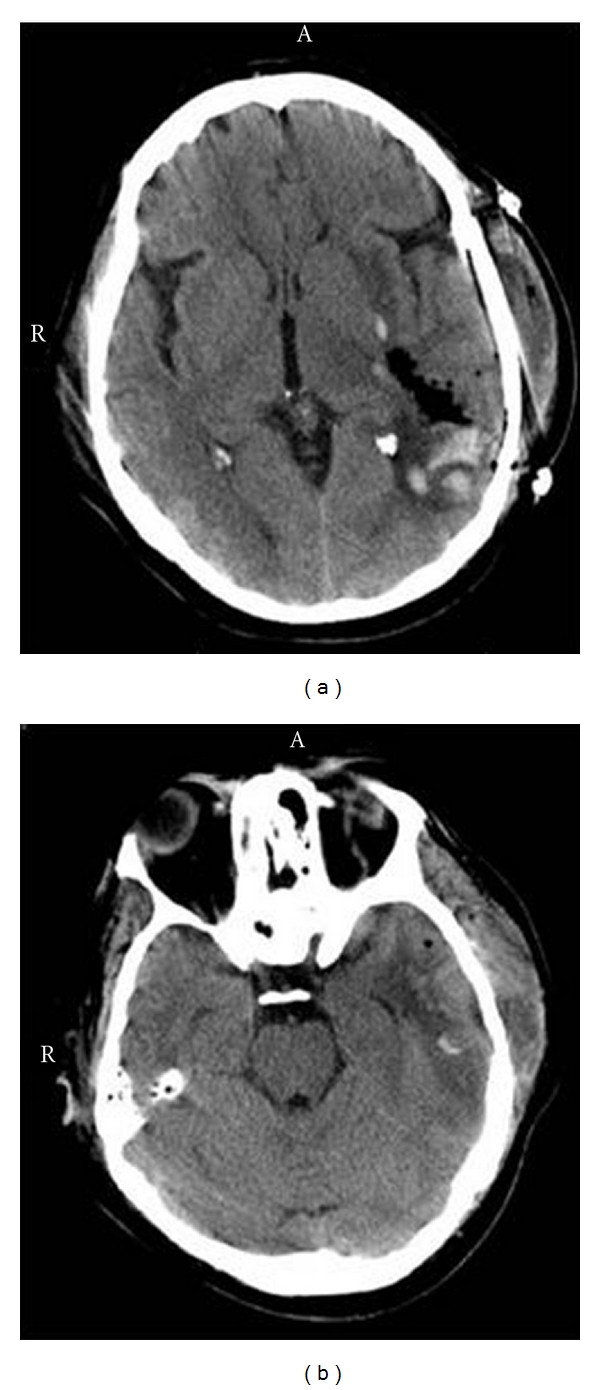
Participant no. 1 with a left temporoparietal lesion. The CT image in (a) depicts an axial scan through the maximum width of the lesion in the left angular gyrus. The dark area within the confines of the left hemisphere shows surgical evacuation of infarcted tissue within the left temporoparietal cortex. The CT image in (b) depicts the maximal width of the lesion in the left inferior temporal lobe. According to neuroradiological convention left side of the image is the right hemisphere and the right side of the image is the left hemisphere.

Participant 1 was a 57-year-old right-handed female with one year of college that had sustained a severe left temporoparietal stroke as a result of left middle cerebral artery occlusion (Figure 1). She was assessed 10 months after stroke, and her initial premorbid FSIQ was estimated to be in the average range (104). She showed resolution of an initial global aphasia into a persistent Broca's aphasia with relative preservation of mathematical reasoning and constructional praxis. She demonstrated reduced verbal concept formation, impaired immediate and delayed retrieval of verbal memory, and impaired immediate nonverbal memory. Executive functions were also poor. Since participant 1 suffered a large dominant hemisphere perisylvian stroke the composite WAIS-IV scales did not appear to be an accurate estimate of her actual level of function. Thus, the WAIS-IV subtest scales were interpreted in the context of an abbreviated test of general intellectual functioning [51].

Participant 1's scores on the WAIS-IV were all far below expected values consistent with sustaining a large perisylvian dominant hemisphere stroke [52] with the exception that Figure Weights was in the normal range (Table 2). The Block Design subtest was the second highest score, and this task has been shown to be especially sensitive to right posterior lesions which in participant 1's instance were distally located from the left perisylvian lesion and undamaged [53]. Consonant with the lateralized nature of her stroke lesion, there was an almost a one standard deviation unit difference in favour of the Perceptual Reasoning Index subtests compared to the Verbal Comprehension Index subtests. Thus, participant 1 demonstrated a pervasive pattern of linguistic deficits with pronounced residual Broca's aphasia. 

Given these multiple deficits, the conclusion can be drawn that the normal range score for Figure Weights implies that the left temporoparietal junction is not likely to be critically involved in performance on this subtest. In the absence of an impaired effect for Figure Weights by participant 1, a further inference would be that Figure Weights could well be more heavily dependent on the integrity of neural substrates distributed within the right hemisphere. Visual Puzzles was in the impaired range for participant 1 implying that the some aspects of intact left hemisphere functioning are indeed necessary for adequate performance on this task. Cancellation was similarly impaired, suggesting that the left temporoparietal junction could be necessary for performance on this subtest.

Participant 1's K-BIT score using the Matrices composite was observed to be 84 which was within the normal range. However, this score was still below expected values. Such a discrepancy would be consistent with sustaining a severe dominant hemisphere stroke. Participant 1's Immediate Auditory and Visual Memory on the WMS-IV were both in the impaired range (both *T*'s < 24—using *T*-scores with a mean of 50 and a standard deviation of 10). In contrast, Delayed Auditory Memory (*T* = 20) was disproportionately adversely affected compared with Delayed Visual Memory (*T* = 30). Auditory and Visual Recognition on the WMS-IV were both in the average range implying normal aural and visual encoding processes. Participant 1 was impaired on Sentence Repetition and Fluency and normal on Comprehension of the Multilingual Aphasia Examination—Third Edition [54] consistent with a diagnosis of residual Broca's aphasia. Participant 1 was also impaired on Color Trails (*T* < 20) implying impaired sustained attention and sequencing [55], and she was also selectively impaired on Finger Tapping (*T* = 13) and Grip Strength (*T* = 32) with the right hand only [43]. The motor deficits again are consistent with sustaining a large dominant hemisphere lesion.

### 3.2. Participant 2: Left Frontal Lobe Lesion

**Figure 2:**
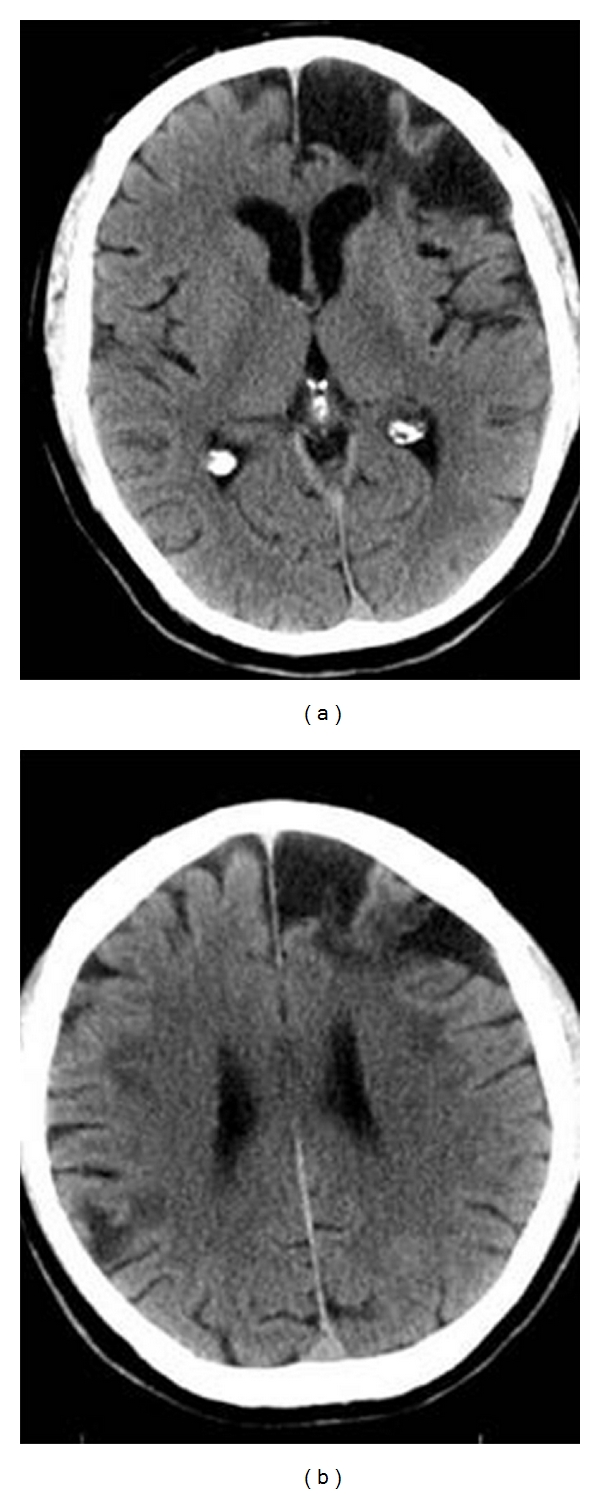
Participant no. 2 with a left frontal lobe lesion. The CT image in (a) depicts the extensive left inferior frontal lobe lesion occurring as a result of an anterior communicating artery aneurysm. The CT image in (b) depicts the same lesion extending contiguously to include the infarcted tissue in the left anterior superior frontal lobe.

Participant 2 was a 64-year-old right-handed male with five years of college education that had sustained a large infarct in the left inferior frontal lobe as a result of an anterior communicating artery aneurysm (Figure 2). He was assessed 31 months after stroke, and his premorbid FSIQ was estimated in the high average range at 117. Participant 2 demonstrated poor verbal abstraction, verbal working memory impairment, poor verbal sequencing, poor auditory memory, poor olfactory discrimination, verbal proactive interference, verbal perseveration, and poor executive functions.

Participant 2 performed significantly higher on the Processing Speed Index compared to the Verbal Working Memory Index (*P* < .05) see Table 3. Verbal working memory has been previously shown to involve left lateral prefrontal and left parietal neural substrates [56], and consistent with these findings, portions of the left superior prefrontal cortex were unambiguously damaged in participant 2 (Figure 2). 

Information was a significant strength compared with the mean of all the 15 subtests attesting to participant 2's better-than-average level of education on a subtest known to be largely resistant to the effect of brain damage [34]. Similarities and Comprehension were significantly below expected values consistent with sustaining a large left inferior frontal lobe lesion. Rzechorzek found that left frontal lesions impaired performance more than right frontal lesions on Similarities [57], and Lezak notes that Comprehension is vulnerable to lesions broadly within the left hemisphere [34, page 630]. Despite a large left frontal lesion, participant 2 showed completely spared Figure Weights performance (in a manner similar to participant 1 with a left hemisphere lesion) hinting at a lack of a necessity of intact left frontal regions for adequate performance on this task (Table 4). 

Although Digit Span was within the normal range Digit Span Sequencing was significantly below Digit Span Forward and Backward. Such deficiencies in verbal sequencing are routinely found after left frontotemporal types of lesions more generally [48]. Finally, performance on the Cancellation subtest was poor which is inconsistent with the usual finding that right posterior lesions disproportionately adversely affect such visual search tasks [25]. In the WAIS-IV Cancellation subtest the requirements are that participants match colors (red, yellow, orange and blue) with forms (star, circle, square, and triangle) in order to search for a target amidst the distractors. The salience of object features such as shape and color suggests that object-based attentional mechanisms may be operative. Previous studies have shown that object-based attention is usually more sensitive to left hemisphere lesions [58].

On the WMS-IV, Visual Immediate Memory (92) was significantly better than Auditory Immediate Memory (52) consistent with the left-sided lesion. Participant 2 was significantly impaired on the Brief Test of Attention (*T* = 30) which is a test of auditory divided attention [37]. Participant 2 was significantly impaired on the Category Fluency Test (*T* = 30) which is a semantic fluency task [59]. He was also impaired on the number of categories achieved (*T* < 28) on the Wisconsin Card Sorting Test [41]. Participant 2 was impaired on the Booklet Category Test (*T* = 30) which is a measure of nonverbal concept formation [42]. Participant 2 scored below the 5th percentile on the Smell Identification Test perhaps consonant with localization of part of the lesion into the left orbitofrontal cortex [44]. He performed in the impaired range on Finger Tapping Test (*T* = 33) with his right hand consistent with the neuroanatomical extent of his left frontal lesion.

Participant 2 was assessed with The Awareness of Social Inference Test (TASIT) given that lateralized frontal injuries have been associated with impairments in emotion and mood regulation as well as changes in personality [46, 60]. He was impaired on all three parts of the TASIT (all *T*'s < 29) which requires the recognition of emotional expressions in audiovisual vignettes depicting interactions between two people. Participant 2 was specifically impaired in the recognition of the emotions of happiness, anxiousness, and disgust. He was also impaired in interpreting sarcasm as well as detecting whether one member of the interacting videotaped dyad was trying to deceive the other through lying. Finally, participant 2 scored in the impaired range on the composite scale of Conscientiousness on the NEO Personality Inventory Revised [61]. Family members corroborated that this heightened impulsivity was a significant adverse personality change after stroke. Left frontal lesions and frontal lesions extending into the orbitofrontal cortex have been previously shown to be apt to cause increased impulsivity [50]. 

### 3.3. Participant 3: Right Frontal Temporal Lobe Lesion

**Figure 3:**
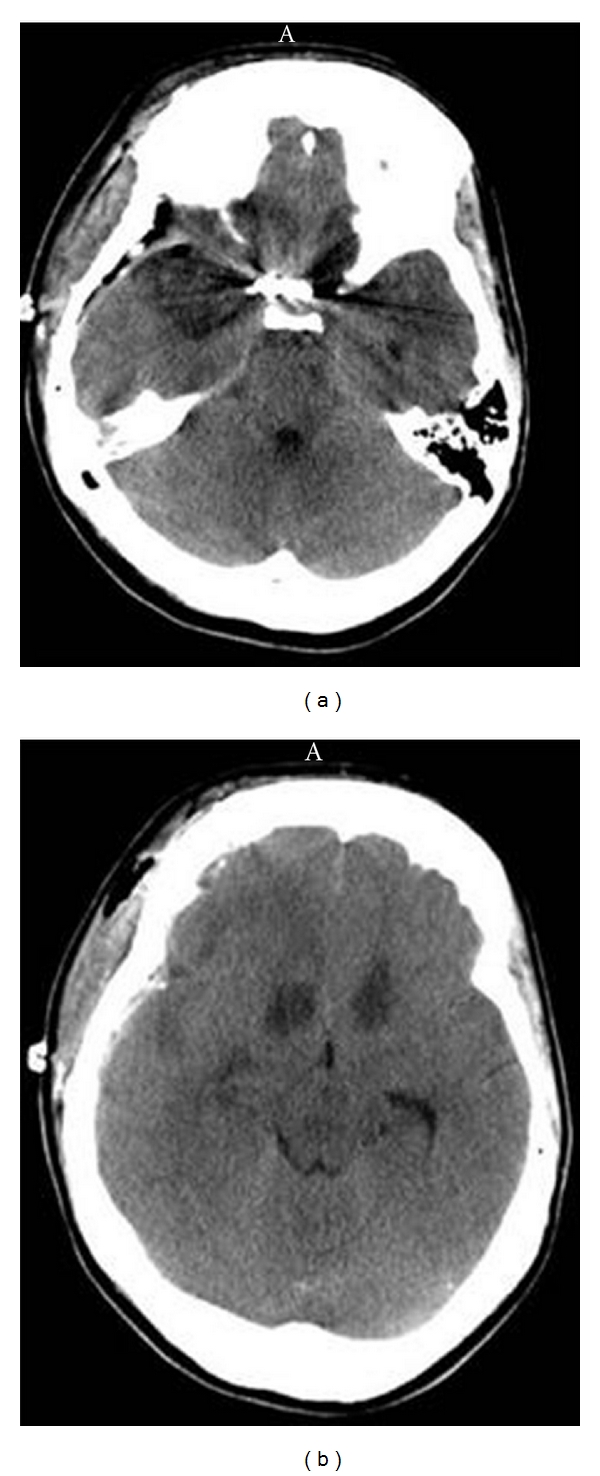
Participant no. 3 with a right frontotemporal lesion. The CT image in (a) depicts a large lesion within the right anterior inferior medial temporal lobe with incursion of the lesion to include both straight gyri of the orbitofrontal lobes. The CT image in (b) depicts bilateral lesions in the cortex underlying the anterior-inferior cingulate gyri. Although the lesions are bilateral, (and this poses some degree of difficulty in making firm conclusions), the lesions are significantly larger and of greater extent in the right hemisphere.

Participant 3 was a 35-year-old right-handed female with five years of college that had sustained a predominately right inferior frontal-temporal lobe localized infarct after an anterior communicating artery aneurysm (Figure 3). She was assessed 19 months after stroke, and her premorbid FSIQ was estimated to be in the superior range at 123. Participant 3 demonstrated a material-specific deficit in auditory memory with some evidence of postmorbid development of emotional disinhibition. 

Participant 3 performed significantly better on the Verbal Comprehension Index compared to the Verbal Working Memory Index and the Processing Speed Index (both *P*'s <.05) The Perceptual Reasoning Index was also significantly higher than the Verbal Working Memory Index (*P* < 0.05) (Table 5).

Matrix Reasoning and Figure Weights were significant strengths for participant 3 and Digit Span and Symbol Search were significant weaknesses. The superior level performance on the Figure Weights subtest despite a large infarct within the right anterior temporal lobe suggests that the ventral stream within the right hemisphere is unlikely to be involved in performance on this subtest. Similarly, scores in the intact range on the Cancellation subtest for participant 3 is concordant with the hypothesis that the right ventral stream is unlikely to be involved in performance on this task either. Previous studies have shown an absence of activation in right anterior temporal regions during functional neuroimaging of various visual search tasks akin to Cancellation. Rather, the right inferior parietal lobule is consistently activated by conventional visual search tasks [62]. Again, it is important to stress that Figure Weights was in the superior range suggesting that right-sided ventral stream neural circuits cannot be involved in performance on this task. Visual Puzzles was in the normal range for participant 3 suggesting that right ventral stream neural substrates may not be involved in performance on this subtest (Table 6).

Participant 3's poor relative performance on Digit Span appears to have been largely attributable to poor performance on Digit Span Forward which was at the fifth percentile. Digit Span Forward measures what is more commonly referred to as the efficiency of attention or short-term auditory memory [34, page 359]. There was a material-specific strength in Visual Immediate Memory (114) and Visual Delayed Memory (109) compared with Auditory Immediate Memory (92) and Auditory Delayed Memory (86) for participant 3 on the WMS-IV. Normally one might expect lower auditory memory scores for a left posterior lesion and lower visual memory scores with right posterior lesion. This reversed pattern of scores for auditory and visual memory could be evidence for bilateral representation of spatial and linguistic functions which some research suggests is more prevalent in women compared to men [52]. Alternatively, some degree of cortical remapping of these neural substrates of memory as a direct consequence of the severity of the injury and assessment almost two years after stroke, could explain these unexpected findings [34]. Participant 3 also performed poorly on the Booklet Category Test which is a test of nonverbal concept formation (*T* = 34).

Participant 3 scored poorly on the Object Decision subtest of the Visual Object and Space Perception Battery [45]. For the Object Decision subtest, each card (*N* = 20) shows four silhouettes containing the shape of one real object and three distracter items (nonsense shapes). The real objects are, thus, nameable, whereas the nonsense shapes are not. The examinee is asked to point out the real one, and the number of correct choices is recorded. Previous research has shown that right ventral stream lesions were sensitive to performance levels on this object decision task [63] and that these types of tasks provides a measure of the integrity of presemantic perceptual systems. Finding elements of an associative agnosia with a large right anterior temporal lesion would be expected [34] and is concordant with the hypothesis that the right hemisphere ventral stream codes nonverbal gestalt aspects of objects like contour for class inclusion and categorization. Hence participant 2's performance on the Object Decision task met the criteria for a diagnosis of associative agnosia. This finding provides a rationale and additional support for the hypothesis that Figure Weights' dorsal spatial working memory task demand characteristics (age-scaled score = 15) do not have any reliance on the right hemisphere's ventral stream object recognition functions. 

### 3.4. Participant 4: Right Thalamus Lesion

**Figure 4:**
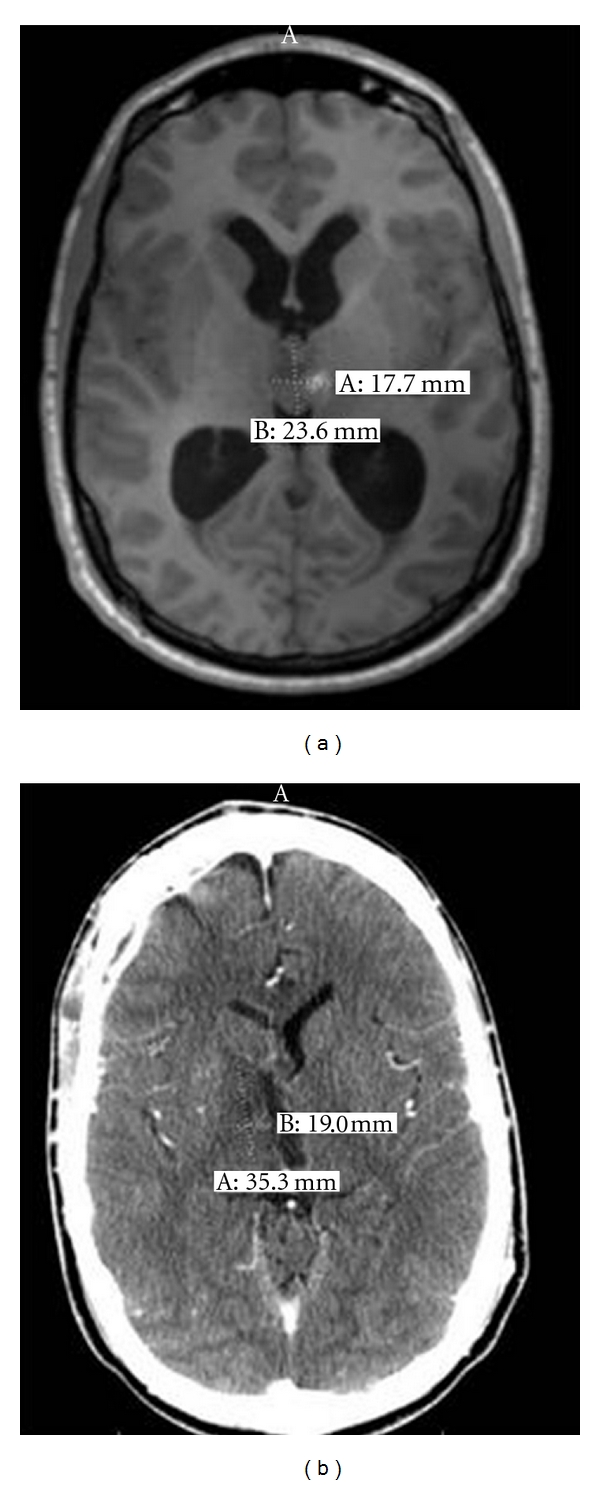
Participant no. 4 with a right thalamic lesion. The MRI image in (a) depicts a calcified third ventricular mass occluding the third ventricle which was subsequently resected. Lateral ventricular dilation is evident. The CT image in (b) depicts resolution of ventricular dilatation subsequent to tumour resection and reopening of the cerebrospinal fluid cisterns. However, (b) shows a right posterior thalamic ischemic infarct appearing two weeks after resection of the third ventricular tumour.

Participant 4 was a 23-year-old right-handed male with 5 years of college that had sustained a right posterior thalamic infarct after a third ventricle tumor (Figure 4). He was assessed four months after infarct, and his premorbid FSIQ was estimated to be in high average range at 114. Participant 4 demonstrated graphomotor slowing, executive function impairment, left hand incoordination, impaired recognition of the emotions of surprise and fear, acquired anosmia, and associative visual agnosia. Participant 4 performed significantly higher on the Verbal Working Memory Index compared with the Processing Speed Index and Perceptual Reasoning Index (both *P*'s <.05) (see Table 7).

For participant 4 Figure Weights was a significant strength compared to Picture Completion, Block Design, and Matrix Reasoning. Block Design, Picture Completion and Coding were all significant weaknesses. These results imply that the normal range Visual Puzzles and Figure Weights aged-scaled scores, (although perhaps dependent on the integrity of right hemisphere) do not require the coordinative functions of the right thalamus. It would appear that performance levels on Cancellation similarly are not dependent on the interaction of the right thalamus based on participant 4's data (Table 8). The WMS-IV Visual Working Memory (70) was significantly less than the WAIS-IV Verbal Working Memory Index (102) consistent with the right thalamus's integral role in visual working memory [64]. Participant 4's performance on the written version of the Symbol Digit Modalities Test was impaired (*T* = 22) consistent with graphomotor slowing [38]. Participant 4 was also impaired on the Ruff Figural Fluency Test (*T* = 28) consistent with this test's assay of nonverbal fluency and perseveration [40], a pattern often witnessed after right frontal lesions where there are dense frontothalamic reentrant tracts [65].

Participant 4 scored at chance on the Smell Identification Test and interestingly Sela and colleagues found that olfactory discrimination was selectively impaired after right posterior thalamic lesions [66]. He also demonstrated impaired eye-hand incoordination with his left hand (*T* = 28) on the Grooved Pegboard Test [67]. Participant 4 scored in the impaired range on the Silhouettes subtest of the Visual Object and Space Perception Battery. He performed marginally better on naming the animal silhouettes (8 out of 15) than on naming the inanimate object silhouettes (5 out of 15). Recent studies show that both thalami are involved in synchronization of neural activity across regions of a stimulus object, and thus are critically involved in establishing coherent representations in object recognition processes. Moreover, when pictorial and verbal labels had to be integrated as in the VOSP Silhouettes subtest right thalamic activation was directly implicated as playing a key neural integration role [68].

In the Advanced Clinical Solutions Affect Naming task, the examinee is shown pictures of faces expressing different emotions and is asked to visually identify the emotion being expressed. Participant 4 scored in the impaired range on Affect Naming (*T* = 33). He scored 0/3 on interpreting surprise and 0/4 items on interpreting fear. Accurately interpreting visual presentations of fear in faces has been previously associated with activation of broad neural networks critically containing the right thalamus [69]. Participant 4 was administered the WMS-III Faces I and II assaying immediate and delayed memory for faces [70]. His performance was completely normal and he showed no evidence of prosopagnosia. Participant 4 scored in the normal range on all three parts of The Awareness of Social Inference Test. Since the TASIT consists of audiovisual vignettes as opposed unimodal sensory stimuli such as visual-only faces, (on the Advanced Clinical Solutions Social Perception battery), it appears that multimodal audiovisual meaning-based integrative functions have not been adversely affected by his right thalamic lesion. Multimodal audiovisual motor integration of facial and vocal emotion has usually been associated with the integrity of the right superior temporal sulcus [71] which was entirely spared in participant 4's instance.

### 3.5. Participant 5: Right Temporoparietal Lesion

**Figure 5:**
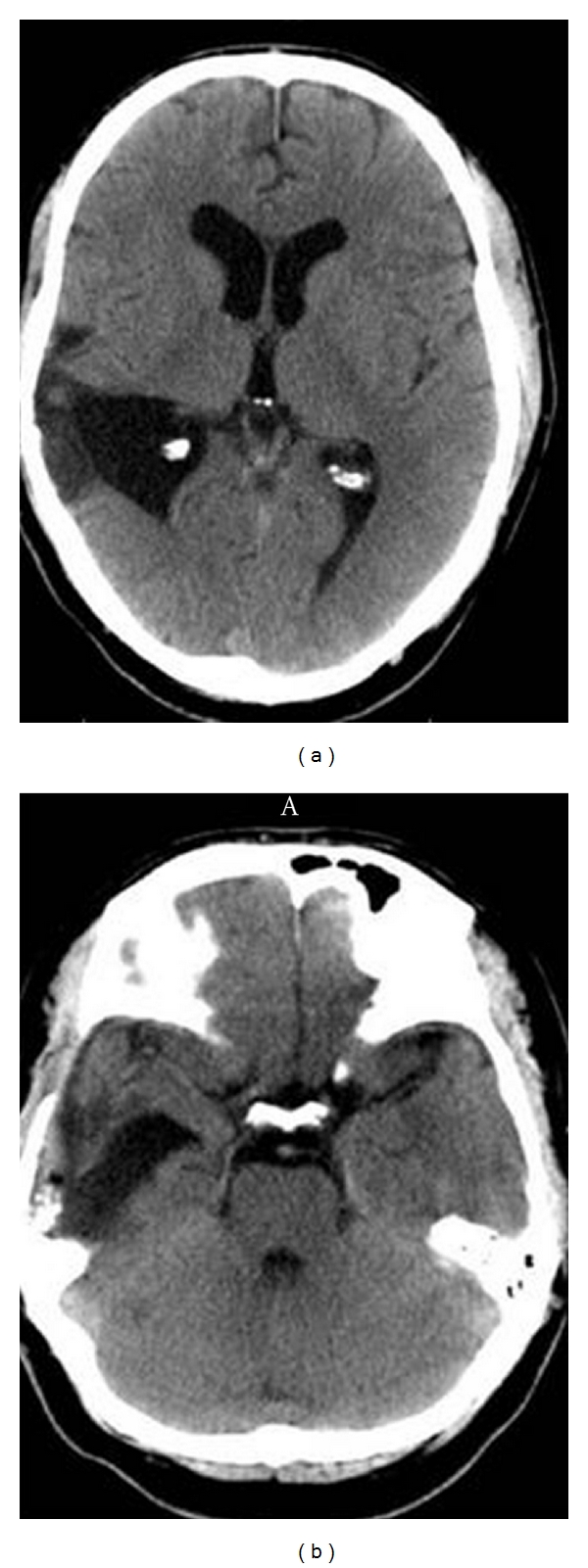
Participant no. 5 with a right temporoparietal lesion. The CT image in (a) depicts severe damage to the right inferior parietal lobe and right temporoparietal junction. The CT image in (b) shows the downward extension of the lesion into the right superior temporal gyrus. The dark areas in (a) and (b) panels illustrate evacuation of infarcted brain tissue by neurosurgery.

Participant 5 was a 40-year-old left-handed male with two years of postsecondary technical training who sustained a large right temporoparietal lesion (Figure 5). He was assessed 8 months after injury, and his premorbid FSIQ was estimated to be in the average range at 103. Participant 5 demonstrated elements of an acquired nonverbal learning disability as well as a problems in performing mathematical operations. He also showed a visual working memory deficit and visual delayed memory impairment. Participant 5 showed a sustained and selective visual attention impairment. He demonstrated specific difficulties in self-monitoring and implementation of strategies in the nonverbal domain. Participant 5 showed elements of a pragmatic language understanding impairment. He also was impaired in the comprehension of facial expressions and prosody. Finally, participant 5 demonstrated constructional apraxia. 

There was a large discrepancy between participant 5's estimated premorbid FSIQ of 103 derived from the Test of Premorbid Functioning and his observed FSIQ of 71 obtained on the WAIS-IV. This discrepancy attests to the large size and adverse effect of this large right temporoparietal lesion on general intellectual functioning. Right parietal lesions have been consistently been shown to result in the largest decrements in full-scale IQ [34, 53] (Table 9). 

Information, Matrix Reasoning, Figure Weights, Picture Completion, Arithmetic and Symbol Search were all below expected values. Digit Span was a significant strength for participant 5. Digit Span Forward was significantly better than Digit Span Backwards. Lezak (1995) notes that participants with large lesions are likely to perform exceptionally poorly on Digit Span Backwards [34, page 368]. Visual Puzzles was intact despite a large right temporoparietal lesion suggesting that Visual Puzzles relies to a certain extent on cognitive processes taking place in the intact left hemisphere. In contrast, Figure Weights was severely impaired implying a strong dependence on right hemisphere networks for this subtest (Table 10).

Finally, Cancellation was unimpaired despite the large and extensive right parietal lesion. The latter finding could potentially be explained by the fact that this left-hander had some degree of preexistent mixed dominance of spatial and linguistic functions [27]. Given that intraparietal sulci of the parietal lobes are routinely activated during conjunctive search [58] and that the critical right temporoparietal region was unambiguously damaged the results suggest the examinee performed the task using other cognitive mechanisms. Cancellation involves conjunctions between shape (square and triangle) and color (red and yellow) in trial 1 and shape (star and circle) and color (orange and blue) in trial 2. The pattern of results would be well-accounted for by use of a verbal labelling strategy, if for instance the subject focused on the red square and yellow triangle in trial 1 and the orange star and blue circle in trial 2. 

This account seems very likely for three reasons. Firstly, the temporal duration of each item is only 45 seconds and the stimulus sheet consists of a single page. Practice items ensure that the examinee has properly understood the task and encoded the two target items. Secondly, the examiner rehearses with the examinee the target shape and color conjunction (e.g., red square and yellow triangle for item 1) no less than four times before the presentation of each of the two search arrays. Finally, the examinee only needs to locate 7 items in each visual field (left, right) on each of the two items in order to receive a Cancellation score within the average range. With such a short time on task (1 minute and 30 seconds total) and the limited number of targets that are required for average performance, it seems that this lowest loading g-factor subtest (0.37) could well function as a screening instrument for visual neglect. 

Participant 5 also demonstrated a significant strength in Auditory Delayed Memory (95) compared with Visual Delayed Memory (75) as measured by the WMS-IV. The Visual Working Memory Index (63) obtained on the WMS-IV was significantly below the Verbal Working Memory Index (82) obtained on the WAIS-IV consonant with damage to right frontoparietal visual working memory networks [22]. Participant 5 demonstrated a significantly impaired performance on Visual Sustained and Selective Attention as measured by the Ruff 2&7 Selective Attention Test [36]. Damage to right inferior parietal neural substrates would be naturally expected to result in impaired sustained and selective visual attention [39]. His performance on the D-KEFS Verbal Fluency Test [51] was significantly impaired (*T* = 28) implying poor phonemic fluency. Participant 5's performance on the Ruff Figural Fluency Test (*T* = 28) was significantly impaired in terms of nonverbal fluency [40]. He was also severely impaired on the Booklet Category Test (*T* = 12).

Participant 5 scored in the impaired range on the Object Decision subtest (*T* = 2). In the Object Decision task of the Visual Object and Space Perception battery only the gestalt aspects of the shadowed stimulus and not features specific to a class of object are utilized in identification [72]. This test would, thus, be expected to be especially sensitive to right hemisphere lesions. The Silhouettes subtest of the Visual Object and Space Perception battery uses three-dimensional shadow images in which participants are required to name animate or inanimate objects in unusual views. Object recognition thresholds were first calibrated in the stimulus set in terms of difficulty through a specific angle of rotation of the shadowed image. Participant 5 scored in the extremely low range on the Silhouettes subtest (*T* = 18). Both tasks are similar to tasks used to diagnose apperceptive agnosia [15] and given the extension of the lesion into the right inferior occipitotemporal cortex these findings are perhaps not unexpected.

Participant 5 scored in the impaired range (*T* = 30) on the Affect Naming subtest illustrative of visual modality-specific deficit in understanding facial expressions. In the Prosody-Face Matching task the examinee listens to audio of a voice stating a stimulus sentence. The examinee is then shown a page with six faces expressing different emotions and is asked to point to the face that matches the emotional tone of the speaker. Prosody-Face Matching requires the cross-mapping of audio and visual cues in emotional expression identification. Participant 5 scored in the impaired range (*T* = 34) on Prosody-Face Matching subtest suggestive of difficulties in integrating audiovisual cues. Finally, in the Prosody-Pair Matching task, the examinee hears a statement and, using the prosody from the audio, selects the picture that best matches the expressed statement. Pictures show two people interacting using body language and facial expressions. This subtest emphasizes some of the higher level pragmatic aspects of emotional language comprehension in dyads. Participant 5 scored in the impaired range on this subtest (*T* = 26). Pragmatic aspects of language are heavily dependent upon the integrity of the right hemisphere and the right temporoparietal junctions especially [73].

## 4. Discussion

In this study, five patients with large strokes or cortical excisions were evaluated with the Wascana Rehabilitation Centre's Standard Neuropsychological Battery. The administered tests included the WAIS-IV, WMS-IV, and the Advanced Clinical Solutions battery. Participants were also examined with a selection of executive function and attention measures as well as tests of sensory and motor functions. All five of the patients' lesions were well characterized at a coarse enough level to coincide well with specific hypotheses of Milner and Goodale's model of the two streams of processing in the human brain [47]. More importantly, each of the cognitive neuropsychological profiles of the five patients were congruent with previously described neuropsychological syndromes in which lesions limited to regional brain areas would be expected to have deleterious effects on specific cognitive functions. This careful delineation of not only expected neuropsychological impairments (but also the absence of neuropsychological signs and symptoms when there were no lesions) in critical brain areas provides criterion-related validity to our double dissociation methodology (Table 11).

The literature review suggests that long-range frontocerebellar tracts emanating from right hemisphere premotor cortex and decussating across to the left cerebellum are likely to be important for optimal performance on the Visual Puzzles subtest [10]. Linn and Petersen demonstrated that such mental rotation tasks can be solved by both visualization and verbal strategies suggesting bilateral hemispheric processing [12]. Finally, mental rotation tasks previously demonstrated reliance on bilateral posterior parietal cortex and bilateral premotor cortex along with posterior occipital cortices [14, 15]. Such patterns of activation as well as the spatial nature of rotations, (which are temporally graded in terms of degree of rotation), [17] suggest use of dorsal stream online nonverbal transformation. Our data showed that left temporoparietal lesions had the largest effect on mental rotation on the Visual Puzzles task. Participant 2's left prefrontal lesion did not adversely affect performance on Visual Puzzles consonant with premotor cortex being the most rostral extent of cortex that would be activated in this type of task. Participant 3's right temporal lesion similarly did not adversely affect performance suggesting that in this predominately nonverbal task it is the dorsal stream premotor-parietal tracts that are most critical for performance on Visual Puzzles.

**Figure 6:**
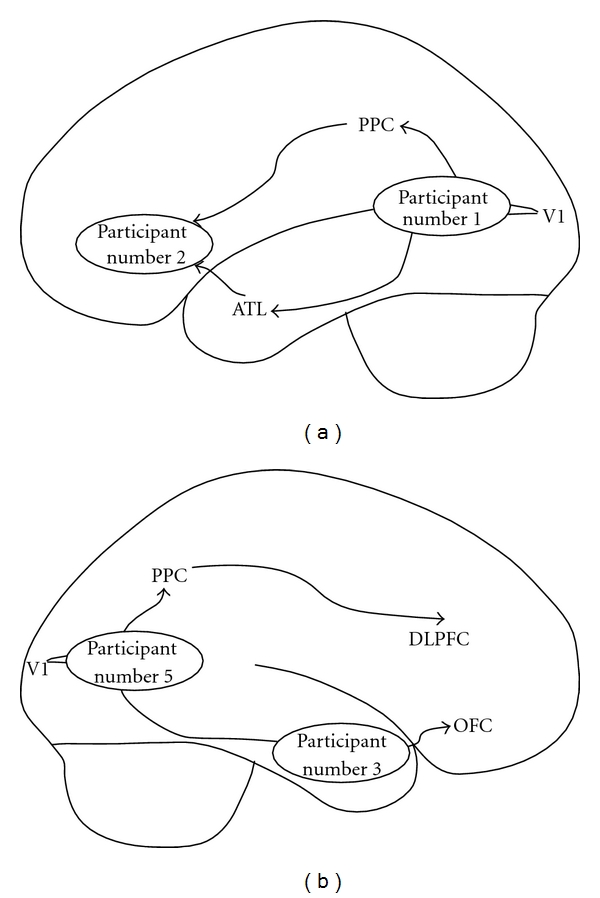
Topography of participant's brain infarcts conceptualized in terms of Milner and Goodale's theory of ventral and dorsal streams [47]. (a) depicts the left hemisphere view of the distribution of lesions in the cortex, whereas (b) illustrates the distribution of lesions in the right hemisphere. Note that participant no. 4 with a right posterior thalamic lesion is not depicted. Legend: PPC: posterior parietal cortex, V1: primary visual cortex, OFC: orbitofrontal cortex, ATL: anterior temporal lobe, and DLPFC: dorsolateral prefrontal cortex.

Visual Puzzles was unaffected by Participant 4's right thalamic lesion consistent with neuroimaging studies showing that the thalamus is not necessary for mental rotation [14]. Finally, despite a large right temporoparietal lesion Visual Puzzles was not significantly impaired in Participant 5, although performance levels could have been attenuated. The literature review would suggest that bilateral posterior parietal lesions ought to be expected to adversely affect performance on Visual Puzzles. Rather the results imply that Visual Puzzles shares some characteristics with predecessor tasks yet possesses unique cognitive properties compared to many conventional mental rotation tasks (Figure 6). 

In fact, in Visual Puzzles, examinees must mentally transform images of multiple shapes instead of single shapes or volumes. Characteristically, also these multiple rotations must be undertaken in an optimal sequence in order to educe the overall gestalt target. The “jigsaw pattern” eduction process implies a degree of correct sequencing of successive rotations. Spatial sequencing of successive movements is particularly prone to lesions of the supramarginal gyrus in the left hemisphere [74] and indeed in participant 1 the left supramarginal gyrus was damaged. Nonetheless, the second lowest score on Visual Puzzles was for participant 2 with a large left prefrontal lesion with perhaps some degree of extension of the infarct into the left inferior lateral premotor cortex. Taken together, the results suggest that bilateral premotor, bilateral posterior parietal and left cerebellar regions are involved in performance on Visual Puzzles and that the left temporoparietal cortex may particularly play a special role. 

Based on the literature review Figure Weights should be dependent upon those neural substrates previously shown to be involved in performance of pure tasks of fluid reasoning [6] such as lateral prefrontal cortex, posterior parietal cortex, dorsal anterior cingulate, and lateral posterior cerebellum. All of these regions have been shown to critically involve executive attentional control [19]. Relatedly, quantitative reasoning does not necessarily have to involve the left hemisphere. Instead lesion studies show that mathematical reasoning can be undertaken by adult patients with large left hemisphere lesions suggesting that the right hemisphere at least in adults has the potential to fully instantiate these cognitive processes [20]. Figure Weight's crucial cognitive processes are posited to rely on mathematical syntactic mechanisms that parallel syntax in the left hemisphere. 

Fluid intelligence is usually associated with spatial working memory and the right hemisphere, implying that the quantitative reasoning of the type tapped in Figure Weights predominately involves the right hemisphere [21, 22]. Participant 1 with a very large left temporoparietal lesion was relatively unaffected by the effects of her large stroke lesion. This suggests that quantitative reasoning that is tapped by Figure Weights, (and by extension left hemisphere networks), cannot be dependent exclusively upon verbal processes. Calculation and number processing of a linguistic-mediated nature have routinely been shown in the past to be dependent upon the integrity of the left inferior parietal lobule [75]. However, in Figure Weights, it appears that numerical magnitude estimation and proportional reasoning are the cognitive components differentiating it from previous conventional verbal mathematical processes.

Similarly, a large left frontal lobe lesion (participant 2) had no effect on performance of Figure Weights suggesting that it could be reliant on predominately right lateralized neural substrates. Based on the classification of participant's scores as normal or impaired, we can deduce that Figure Weights is critically dependent upon neural substrates in the right hemisphere. In the context of participants 1 and 2, we can further determine that Figure Weights is not dependent on the right ventral stream pathway based on the superior performance of participant 3 (e.g., age-scaled score = 15). The results of participant 4 rules out the involvement of right thalamic or midbrain structures in performance on Figure Weights. Participant 5's right temporoparietal lesion and impaired performance on Figure Weights suggests critical involvement of the right temporoparietal junction in this subtest. The overall pattern of results strongly implies the involvement of right frontoparietal networks involved in spatial working memory and executive attentional control. Previous functional neuroimaging studies have shown the involvement of right intraparietal sulcus and right prefrontal circuit in number comparison tasks [76] and the right parietal area during the manipulation of numerical quantities [77]. Since Figure Weight's core task is the estimation and balancing or comparison of numerical and proportional quantities (and not simply establishing object-based equivalencies), the deleterious effects of participant 5's right temporoparietal lesion can perhaps be readily explainable.

The results for the Cancellation subtest were perhaps the most unexpected. Previous research with cancellation subtests has often used marking of achromatic lines or letters in large asymmetric arrays as test stimuli. These types of tasks have usually been shown to be most sensitive to right parietal lesions [23–26, 28, 29] and the resulting left visual neglect appears to be due to underlying white matter damage to the frontoparietal and fronto-occipital tracts in the right hemisphere [30]. In participant 3 with a right temporal lobe lesion, Cancellation was in the unimpaired range consistent with the nonnecessity of activation of the right ventral stream for performance on this type of visual search task. Although the right thalamus is important for visual working memory [64] lesions of the right thalamus in participant 4 did not result in attenuation of performance on the WAIS-IV Cancellation subtest. Woodman and colleagues have shown that targets are stored in visual working memory and used to guide attention during visual search tasks such as Cancellation [78]. These researchers showed that the network of brain areas involved in shifting attention during visual search tasks are able to operate essentially independently of anatomical areas involved in visual working memory (vis-à-vis the thalamus above) only if the identity of a visual search target is stable across time as in Cancellation. The latter finding might explain the lack of an effect of a right thalamic lesion on Cancellation performance.

In participant 5, despite a large right temporoparietal lesion there was no effect on Cancellation performance (e.g., age scaled score = 11). Collectively, the results of participants 3, 4, and 5 suggest that Cancellation is not a typical visual search task with a dependence on the right hemisphere and particularly right parietal substrates. Rather, a review of all of the participant's error patterns on the Cancellation subtest suggested that a verbal labelling strategy was most likely being used. The verbal labelling strategy for performance of the Cancellation task is reinforced by the examiner and the demand characteristics of this particular task—emphasizing conjunctive search of forms and colors. In support of the verbal labelling hypothesis, we found that participant 1 with a left temporoparietal lesion and participant 2 with a left frontal lesion were both impaired on Cancellation as would be expected if reliant on linguistic networks. Moreover, the conjunctive object search-like aspects of the task would again suggest the importance of the left hemisphere for successful performance of the WAIS-IV Cancellation subtest [58]. Given that the target is verbally labelled by the examiner four times before the participant views the actual visual array (red square and yellow triangle for on item 1) and (orange star and blue circle for item 2), it seems highly plausible based on the concordant pattern of results in right and left hemisphere lesioned patients that the participants are using a verbal search strategy. Congruent with these hypotheses were recent studies of color and form categorization as used in Cancellation which resulted in activation in left frontotemporal cortex in a functional neuroimaging task [79].

## 5. Conclusions

The data and literature review suggest that Visual Puzzles appears to be reliant on crossed decussated right hemisphere and left cerebellar networks as well as left parietal regions. Both visual and verbal strategies can be used to perform these mental rotation types of tasks suggesting some degree of dual hemispheric processing. However in Visual Puzzles few if any of the item segments are easily verbally labeled or amenable to use of a verbal strategy. Bilateral posterior parietal and bilateral premotor regions appear to be particularly implicated in performance on these mental rotation tasks. On Visual Puzzles, the data suggested a greater reliance on left as opposed to right temporoparietal regions. A large right temporoparietal lesion had minimal affects on the mental rotation task congruent with the hypothesis that the unique sequencing demands of rotated elements involves the left supramarginal gyrus. Figure Weights has displaced the Block Design as one of the highest g-loaded tests in the WAIS-IV battery and has few if any parallels in the either the psychometric or neuropsychological literatures. Nonetheless, the review concludes that it is a relatively pure measure of fluid intelligence and mathematical reasoning. Fluid intelligence has been shown to involve the lateral prefrontal cortex, posterior parietal cortex, dorsal anterior cingulate and lateral posterior cerebellum critically involved in executive control. 

Based on the literature review and results of this clinical case study, it would appear that Figure Weights involves predominately right hemisphere lateralized spatial working memory substrates involved in fluid reasoning. The performance pattern of participants on Figure Weights suggests a predominance of right frontoparietal networks involved in executive control and spatial working memory. The right inferior parietal lobe would also be expected to be involved in numerical magnitude estimation and/or proportional reasoning. There was little evidence of involvement of ventral stream pathways in either hemisphere for Figure Weights, nor did the data suggest a pivotal role for the left hemisphere for performance on this task. The Cancellation task showed a strong dependence on object-based search mechanisms usually shown to involve neural substrates within the left hemisphere. The pattern of results for Cancellation suggested a limited, if any, role for the right hemisphere in performance on this task contrary to a voluminous literature showing dependence of visual search tasks on intact right parietal networks. Rather the results point to a dependence on object-based search involving conjunctions of color and form that is verbally mediated and dependent on intact left frontotemporal structures. 

The WAIS-IV and WMS-IV which were released in 2008 demonstrate three new subtests that are almost completely unique in terms of the cognitive neuropsychological processes involved in performance. Visual Puzzles with its high ceiling items appears to capture the elusive spatial intelligence factor that has often been viewed as the poor step-sister of the general factor. Figure Weights, with its high loading on the general factor has included numerical magnitude estimation and proportional reasoning and appears to be dependent on right lateralized neural substrates. The Cancellation task shows a reliance on left frontotemporal structures previously shown to be instantiate object-based conjunctive search. The Visual Puzzles, Figure Weights, and Cancellation subtests might then be expected to be good measures of bilateral dorsal stream, right dorsal stream, and left dorsal/ventral stream integrity, respectively, using Milner and Goodale's model. 

Obviously, anatomopsychological correlation often relies on CT scans, which so far as they can be informative about the site and extent of lesions, do not yet possess the level of validity of histological observation post-autopsy. Thus, the voluminous neuropsychological lesion literature has historically been based upon the gold standard of histology which by extension places limits on the generalizability of our findings. As a final note, the WAIS-IV, WMS-IV and Advanced Clinical Solutions Social Perception batteries offer co-normed and comprehensive instruments firmly entrenched in neuropsychological theory. Additionally, these tasks might pose desirable properties for implementation in functional neuroimaging environments, thereby integrating psychometric theory with cognitive neuroscience. Such an ambitious task has not been attempted since the early 1980s in the formative years of cognitive neuroscience [80]. Efforts in this direction would likely benefit from implementation of these tasks using new structural and functional neuroimaging modalities, use of advanced multivariate statistical techniques as well as developments in neuropsychology theory and methods.

## Figures and Tables

**Table 1:** Wascana Rehabilitation Centre's Standard Neuropsychological Battery.

Psychological domain	Specific neuropsychological test and reference
Response validity	Advanced Clinical Solutions Effort Assessment [33]
General ability	Wechsler Adult Intelligence Scale-IV [31]
Premorbid functioning	Advanced Clinical Solutions Test of Premorbid Function [33]
Learning and memory	Wechsler Memory Scale-IV [32]
Attention	Ruff 2 and 7 Selective Attention Test (visual) [36]
Attention	Brief Test of Attention (aural) [37]
Attention	Symbol Digit Modalities Test (graphomotor and oromotor) [38]
Executive functions	D-KEFS Verbal Fluency Test [39]
Executive functions	D-KEFS Trail Making Test [39]
Executive functions	Tower of London^DX^ (Culbertson and Zillmer, 2001)
Executive functions	Ruff Figural Fluency Test [40]
Executive functions	Wisconsin Card Sorting Test [41]
Executive functions	Booklet Category Test [42]
Achievement	Wide Range Achievement Test-IV (Wilkinson and Robertson, 2006)
Sensory and motor function	Grip Strength [43]
Sensory and motor function	Finger Tapping [43]
Sensory and motor function	Grooved Pegboard Test [43]
Sensory and motor function	Smell Identification Test [44]
Spatial perception	Visual Object and Space Perception Battery [45]
Social cognition	Test of the Awareness of Social Inference Test [46]
Social cognition	Advanced Clinical Solutions Social Perception battery [33]
Emotional functioning	Beck Depression Inventory—II (Beck et al., 1996)
Emotional functioning	Beck Anxiety Inventory (Beck, 1993)
Emotional functioning	Beck Hopelessness Scale (Beck, 1993)
Personality	House-Tree-Person Drawing Test (Buck, 1970)
Personality	Minnesota Multiphasic Personality Inventory-2-Restructured Form (Ben-Porath and Tellegen, 2008)

**Table 2:** Description of participant no. 1's WAIS-IV subtest profile.

Participant no. 1 WAIS-IV subtests	Age-scaled score	Mean age-scaled score	Difference	Percentile	Qualitative description
Similarities	1	3.13	−2.13	0.1	Impaired
Vocabulary	2	3.13	−1.13	0.4	Impaired
Information	3	3.13	−0.13	1	Impaired
Comprehension	3	3.13	−0.13	1	Impaired
Block design	5	3.13	1.87	5	Impaired
Matrix reasoning	4	3.13	0.87	2	Impaired
Visual puzzles	4	3.13	0.87	2	Impaired
Figure weights	6	3.13	2.87	9	Normal
Picture completion	4	3.13	0.87	2	Impaired
Digit span	3	3.13	−0.13	1	Impaired
Arithmetic	4	3.13	0.87	2	Impaired
Letter-number seq.	4	3.13	0.87	2	Impaired
Symbol search	1	3.13	−2.13	0.1	Impaired
Coding	1	3.13	−2.13	0.1	Impaired
Cancellation	2	3.13	−1.13	0.4	Impaired

**Table 3:** Description of participant no. 2's WAIS-IV composite scale profile.

Participant no. 2 WAIS-IV composite scales	IQ score	95% confidence interval	Percentile	Qualitative description
Verbal comprehension	92	86–99	30	Average
Perceptual reasoning	80	74–88	9	Low Average
Verbal working memory	79	73–88	8	Very Low
Processing speed	97	89–106	42	Average
Full-scale IQ	83	79–88	13	Low Average

**Table 4:** Description of participant no. 2's WAIS-IV subtest profile.

Participant no. 2 WAIS-IV subtests	Age-scaled score	Mean age-scaled score	Difference	Percentile	Qualitative description
Similarities	5	7.13	−2.13	5	Impaired
Vocabulary	10	7.13	2.87	50	Normal
Information	11	7.13	3.87	63	Normal
Comprehension	5	7.13	−2.13	5	Impaired
Block design	8	7.13	0.87	25	Normal
Matrix reasoning	6	7.13	−1.13	9	Normal
Visual puzzles	6	7.13	−1.13	9	Normal
Figure weights	10	7.13	2.87	50	Normal
Picture completion	6	7.13	−1.13	9	Normal
Digit span	6	7.13	−1.13	9	Normal
Arithmetic	7	7.13	−0.13	16	Normal
Letter-number Seq.	6	7.13	−1.13	9	Normal
Symbol search	9	7.13	1.87	37	Normal
Coding	10	7.13	2.87	50	Normal
Cancellation	2	7.13	−5.13	0.4	Impaired

**Table 5:** Description of participant no. 3's WAIS-IV composite scale profile.

Participant no. 3 WAIS-IV composite scales	IQ score	95% confidence interval	Percentile	Qualitative description
Verbal comprehension	121	114–126	92	Superior
Perceptual reasoning	112	105–118	79	High Average
Verbal working memory	88	81–97	21	Low Average
Processing speed	88	81–98	21	Low Average
Full-scale IQ	107	102–112	68	Average

**Table 6:** Description of participant no. 3's WAIS-IV subtest profile.

Participant no. 3 WAIS-IV subtests	Age-scaled score	Mean age-scaled score	Difference	Percentile	Qualitative description
Similarities	16	10.73	5.27	98	Normal
Vocabulary	14	10.73	3.27	91	Normal
Information	11	10.73	0.27	63	Normal
Comprehension	12	10.73	1.27	75	Normal
Block design	13	10.73	2.30	84	Normal
Matrix reasoning	14	10.73	3.27	91	Normal
Visual puzzles	9	10.73	−1.73	37	Normal
Figure weights	15	10.73	4.27	95	Normal
Picture completion	10	10.73	−0.73	50	Normal
Digit span	6	10.73	−4.73	9	Normal
Arithmetic	10	10.73	−0.73	50	Normal
Letter-number seq.	7	10.73	−3.73	16	Normal
Symbol search	7	10.73	−3.73	16	Normal
Coding	9	10.73	−1.73	37	Normal
Cancellation	8	10.73	−2.73	25	Normal

**Table 7:** Description of participant no. 4's WAIS-IV composite scale profile.

Participant no. 4 WAIS-IV composite scales	IQ score	95% confidence interval	Percentile	Qualitative description
Verbal comprehension	86	80–93	18	Low average
Perceptual reasoning	78	73–86	7	Very low
Verbal working memory	102	94–109	55	Average
Processing speed	72	66–83	3	Very low
Full-scale IQ	79	75–85	8	Very low

**Table 8:** Description of participant no. 4's WAIS-IV subtest profile.

Participant no. 4 WAIS-IV subtests	Ag-scaled score	Mean age-scaled score	Difference	Percentile	Qualitative description
Similarities	7	7.27	−0.27	16	Normal
Vocabulary	6	7.27	−1.27	9	Normal
Information	10	7.27	2.73	50	Normal
Comprehension	6	7.27	−1.27	9	Normal
Block design	5	7.27	−2.27	5	Impaired
Matrix reasoning	6	7.27	−1.27	9	Normal
Visual puzzles	8	7.27	0.73	25	Normal
Figure weights	10	7.27	2.73	50	Normal
Picture completion	4	7.27	−3.27	2	Impaired
Digit span	10	7.27	2.73	50	Normal
Arithmetic	11	7.27	3.73	63	Normal
Letter-number seq.	7	7.27	−0.27	16	Normal
Symbol search	6	7.27	−1.27	9	Normal
Coding	4	7.27	−3.27	2	Impaired
Cancellation	9	7.27	1.73	37	Normal

**Table 9:** Description of participant no. 5's WAIS-IV composite scale.

Participant no. 5 WAIS-IV composite scales	IQ score	95% confidence interval	Percentile	Qualitative description
Verbal comprehension	76	71–83	5	Very low
Perceptual reasoning	74	69–82	4	Very low
Verbal working memory	82	76–91	12	Low average
Processing speed	77	71–88	6	Very low
Full-scale IQ	71	67–77	3	Very low

**Table 10:** Description of participant no. 5's WAIS-IV subtest profile.

Participant no. 5 WAIS-IV subtests	Age-scaled score	Mean age-scaled score	Difference	Percentile	Qualitative description
Similarities	7	6.20	0.80	16	Normal
Vocabulary	6	6.20	−0.20	9	Normal
Information	4	6.20	−2.20	2	Impaired
Comprehension	6	6.20	−0.20	9	Normal
Block design	7	6.20	0.80	16	Normal
Matrix reasoning	4	6.20	−2.20	2	Impaired
Visual puzzles	6	6.20	−0.20	9	Normal
Figure weights	4	6.20	−2.20	2	Impaired
Picture completion	5	6.20	−1.20	5	Impaired
Digit span	9	6.20	2.80	37	Normal
Arithmetic	5	6.20	−1.20	5	Impaired
Letter-number seq.	7	6.20	0.80	16	Normal
Symbol search	4	6.20	−2.20	2	Impaired
Coding	8	6.20	1.80	25	Normal
Cancellation	11	6.20	4.80	63	Normal

**Table 11:** Ipsative profile of participant's age-scaled scores on visual puzzles, figure weights and cancellation.

Ipsative WAIS-IV	Participant's age-scaled WAIS-IV scores				
subtest profiles	1	2	3	4	5
Visual puzzles	4 (Impaired)	6 (Normal)	9 (Normal)	8 (Normal)	6 (Normal)
Figure weights	6 (Normal)	10 (Normal)	15 (Normal)	10 (Normal)	4 (Impaired)
Cancellation	2 (Impaired)	2 (Impaired)	8 (Normal)	9 (Normal)	11 (Normal)
